# Early loss of subchondral bone following microfracture is counteracted by bone marrow aspirate in a translational model of osteochondral repair

**DOI:** 10.1038/srep45189

**Published:** 2017-03-27

**Authors:** Liang Gao, Patrick Orth, Kathrin Müller-Brandt, Lars K. H. Goebel, Magali Cucchiarini, Henning Madry

**Affiliations:** 1 Center of Experimental Orthopaedics, Saarland University, Homburg, Germany; 2 Department of Orthopaedic Surgery, Saarland University Medical Center, Homburg, Germany

## Abstract

Microfracture of cartilage defects may induce alterations of the subchondral bone in the mid- and long-term, yet very little is known about their onset. Possibly, these changes may be avoided by an enhanced microfracture technique with additional application of bone marrow aspirate. In this study, full-thickness chondral defects in the knee joints of minipigs were either treated with (1) debridement down to the subchondral bone plate alone, (2) debridement with microfracture, or (3) microfracture with additional application of bone marrow aspirate. At 4 weeks after microfracture, the loss of subchondral bone below the defects largely exceeded the original microfracture holes. Of note, a significant increase of osteoclast density was identified in defects treated with microfracture alone compared with debridement only. Both changes were significantly counteracted by the adjunct treatment with bone marrow. Debridement and microfracture without or with bone marrow were equivalent regarding the early cartilage repair. These data suggest that microfracture induced a substantial early resorption of the subchondral bone and also highlight the potential value of bone marrow aspirate as an adjunct to counteract these alterations. Clinical studies are warranted to further elucidate early events of osteochondral repair and the effect of enhanced microfracture techniques.

Microfracture (MFX) is an important marrow stimulation treatment option for small symptomatic focal articular cartilage defects^1^. Here, multiple perforations of the subchondral bone plate are introduced with the sharp tip of a microfracture awl, allowing for the migration of mesenchymal stem cells from the subchondral bone marrow cavity into the site of the cartilage lesion. Over time, a fibrocartilaginous repair tissue fills the entire defect^2^. Recently, enhanced techniques of microfracture have been described, aiming to better support chondrogenesis within the cartilage defect^3^,^4^,^5^. Among those techniques, the application of bone marrow concentrate as an adjunct to the microfracture technique has been shown to improve articular cartilage repair in animal models^4^,^6^ and patients^7^,^8^. Fortier *et al*. demonstrated the superiority of concentrated bone marrow aspirate augmented microfracture over microfracture alone in a horse model of acute cartilage repair^4^. Gigante *et al*. provided a case series of 9 patients treated with microfracture and a collagen membrane immersed in autologous bone marrow concentrate, showing clinical improvements and a fibrocartilaginous repair tissue after 1 year^8^. Furthermore, bone marrow aspirate has also been proven to possess a robust osseous regenerative capacity when applied to long bone defects in both animals^9^ and patients^10^. A recent double-blind randomized controlled clinical trial showed that additional application of concentrated blood and bone marrow aspirate to the operative treatment of fifth metatarsal stress fractures led to an earlier fracture union than surgery alone^10^.

The integrity of the subchondral bone is a significant factor for the success of cartilage repair^11^,^12^,^13^. Its components, the subchondral bone plate and the subarticular spongiosa, play a key role in mechanically and metabolically supporting the articular cartilage^14^,^15^. Structural alterations of the subchondral bone such as intra-lesional osteophytes^16^,^17^, subchondral bone resorption^18^ and cysts^16^ have, however, been recently demonstrated to occur both in animal models and in patients following marrow stimulation of cartilage defects^17^. Interestingly, these alterations were reported to emerge already at 3 months in the rabbit model^19^ and at 6 months in patients^16^ following microfracture. Frisbie at al. highlighted the importance of removal of the calcified cartilage layer with retention of the subchondral bone plate on the healing of chondral defects treated with microfracture in horses at 1 year^20^. Moreover, such a debridement of human cartilage defects down to the subchondral bone induced penetrations of the subchondral bone plate^21^, thus creating an access to marrow elements. Hoemann *et al*. proved that new bone formation already started at 2 weeks following the marrow stimulation in a rabbit model, accompanied by a high activity of osteoclasts, serving as cellular mediators of marrow-derived cartilage repair^22^. However, the very early effects of microfracture on the subchondral bone in a large animal model remain unknown to date. Besides, as microfracture requires a meticulous debridement of the calcified cartilage layer down to the subchondral bone plate, the early *in vivo* effect of such debridement also remains unexplored. Finally, to the best of our knowledge, the early *in vivo* effect of bone marrow aspirate enhanced microfracture treatment on the subchondral bone has not been investigated so far in this context.

In the present study, we evaluated the early effects of debridement alone and microfracture treatment without or with bone marrow aspirate on cartilage and subchondral bone repair in a full-thickness chondral defect model in minipigs. We hypothesized that early osteochondral repair following bone marrow aspirate enhanced microfracture would be superior to debridement and microfracture alone. We also tested the hypothesis that additional application of bone marrow aspirate to the microfracture procedure would improve the status of the subchondral bone compared with microfracture alone.

## Results

### Standardized defect creation and treatment

**Figure 1:**
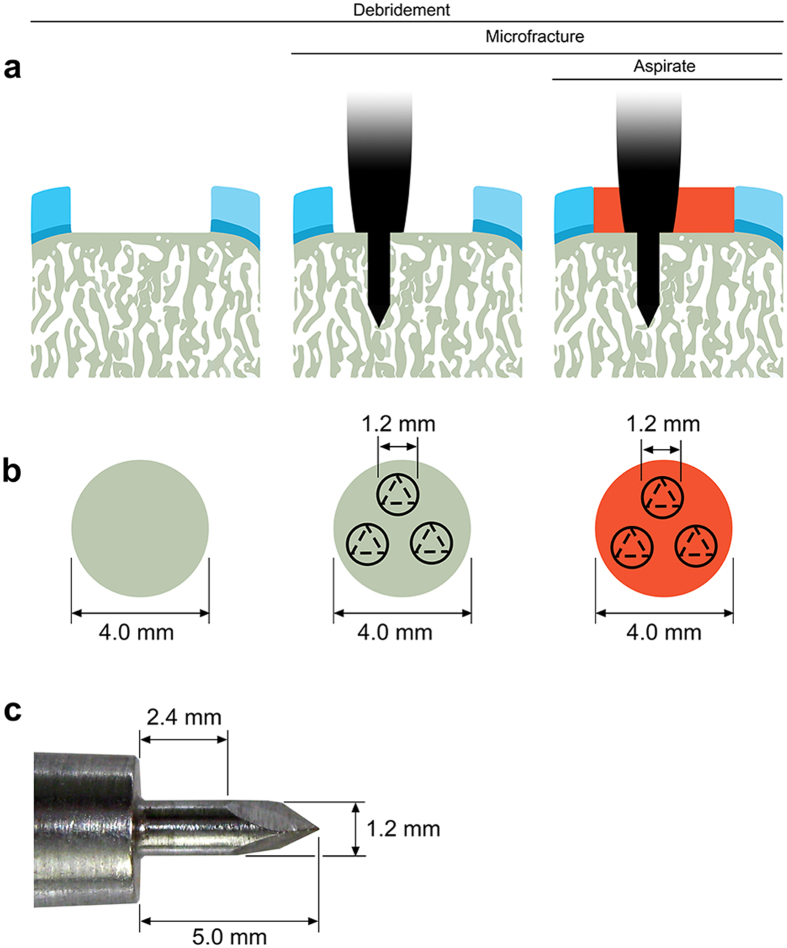
Schematic drawing depicting the study design. Standardized circular (diameter 4 mm), full thickness chondral defects were created in the trochlear facets of the hind legs of minipigs. (**a**) Three treatments were applied including debridement alone (debridement group), debridement and microfracture (microfracture group), and debridement and bone marrow aspirate enhanced microfracture (enhanced microfracture group). (**b**) Top view of defects following the three treatments. (**c**) The trocar-shaped microfracture awl tip consists of a distal trihedral head (length 2.6 mm), a middle cylindrical body (length 2.4 mm, diameter 1.2 mm), and a proximal penetration stop, allowing a standardized penetration depth to 5.0 mm. Articular cartilage: light blue; calcified cartilage: dark blue; subchondral bone: grey; bone marrow aspirate: red.

Uniform circular full-thickness chondral defects were created in the trochlear facets of the hind joints of minipigs (Fig. 1) and always debrided down to the subchondral bone plate. Defects were treated with three strategies including debridement alone (debridement group), debridement and microfracture (microfracture group), and debridement and bone marrow aspirate enhanced microfracture (enhanced microfracture group) in a standardized fashion (Fig. 1a,b). In both microfracture groups, 3 identical microfracture holes were introduced in the subchondral bone of each single cartilage defect with a custom-made microfracture awl (Fig. 1c). Defects in the enhanced microfracture group were additionally covered with a layer of autologous bone marrow aspirate.

### Articular cartilage repair

#### Macroscopic findings

**Figure 2:**
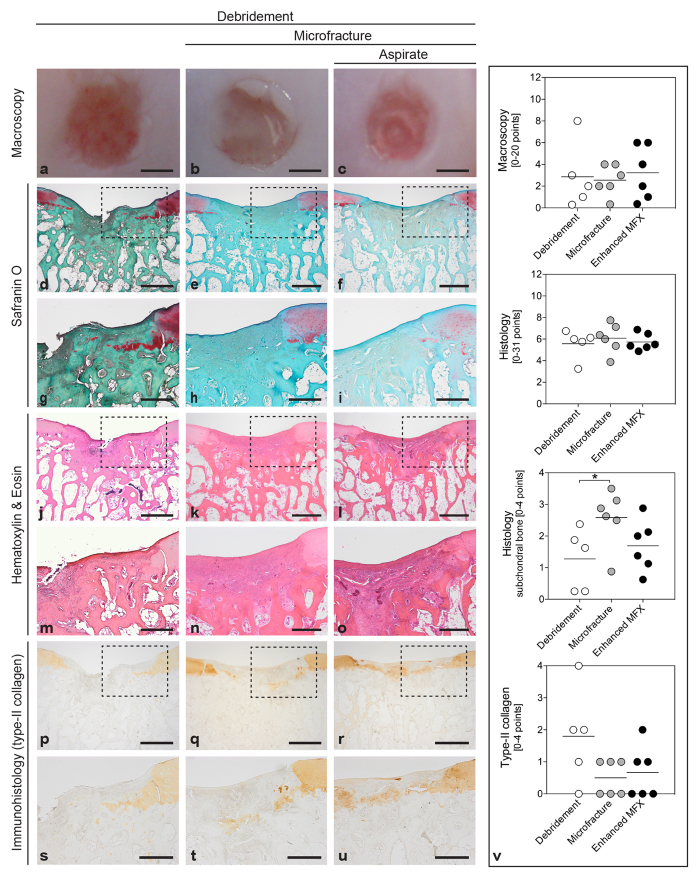
Macroscopic, histological and immunohistochemical analyses of the osteochondral unit. Standardized circular full-thickness chondral defects in the trochlear groove of minipigs were treated by (1) debridement (debridement group), (2) debridement and microfracture (microfracture group), and (3) debridement, microfracture, and application of bone marrow aspirate (enhanced microfracture group). By macroscopic grading^23^, no significant differences in articular cartilage repair were detected between debridement group. (**a**), microfracture group (**b**), and enhanced microfracture group (**c**). According to Sellers *et al*.^24^, histological analysis of the cartilaginous repair tissue stained with safranin O (**d**–**i**) and hematoxylin and eosin (**j**–**o**) also yielded no significant differences between debridement (**d**,**g**,**j**,**m**), microfracture (**e**,**h**,**k**,**n**), and enhanced microfracture group (**f**,**i**,**l**,**o**), except for a significantly higher percentage of subchondral bone within defects treated by debridement alone (v). Immunoreactivity to type-II collagen^64^ was similar between defects treated with debridement (**p**,**s**), microfracture (**q**,**t**), and enhanced microfracture (**r**,**u**). (**g**–**i**,**m**–**o** and **s**–**u**) show the corresponding higher magnification images of the area within the dotted lines in the upper images. Black triangles denote defect borders (**d**–**u**). Scale bars: 2.0 mm (**a**–**c**), 0.5 mm (**d**–**f**,**j**–**l**,**p**–**r**), and 1.0 mm (**g**–**i**,**m**–**o**,**s**–**u**).

During defect preparation, immediate bleeding from the underlying subchondral bone plate was always observed after removal of the calcified cartilage layer in debridement, microfracture, or bone marrow aspirate enhanced microfracture procedure. When the animals were euthanized at 4 weeks postoperatively, no joint effusion, macroscopic inflammation, peri-articular osteophytes, and adhesions were noticed in all treated joints following the three cartilage repair strategies (Fig. 2a–c).

Semi-quantitative macroscopic analysis of articular cartilage repair in each defect according to the score developed by Goebel *et al*.^23^ showed no significant differences between defects from the three groups for all individual parameters and the average total score (debridement: 8.60 ± 6.31; microfracture: 10.67 ± 1.86; enhanced microfracture: 9.50 ± 4.46; *P* = 0.745) (Fig. 2v; Supplementary Table S1).

#### Histological evaluation

Histological evaluation according to Sellers *et al*.^24^ revealed that microfracture neither without nor with application of bone marrow significantly improved the overall aspect of articular cartilage repair compared with debridement alone (0.075 < *P* < 0.896) (Fig. 2d–o,v; Supplementary Table S2). Regarding the single parameter of subchondral bone reconstitution, a significantly impaired score value was observed following microfracture (2.58 ± 1.01) compared with debridement (1.28 ± 0.99; *P* = 0.050) (Fig. 2v). This adverse effect of microfracture on the histological aspect of subchondral bone reconstitution was counteracted by additional bone marrow aspirate (1.69 ± 1.11; *P* = 0.714 for enhanced microfracture *versus* debridement) (Supplementary Table S2).

#### Immunohistochemical evaluation

Immunoreactivity to type-II collagen was not significantly different between the three groups (debridement: 1.80 ± 1.48; microfracture: 0.70 ± 0.52; enhanced microfracture: 0.50 ± 0.55; *P* = 0.072) (Fig. 2p–u,v; Supplementary Table S3).

### Subchondral bone reconstitution

#### Histomorphometric analysis of the subchondral bone within the defect regions

**Figure 3:**
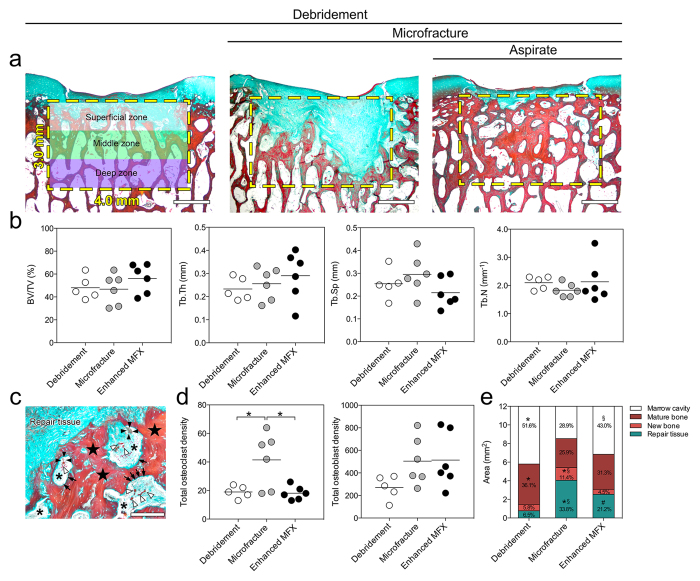
Histomorphometric analyses of subchondral bone changes below the cartilage defects treated with the three experimental strategies. (**a**) Representative images showing the pattern of subchondral bone changes in sections through the central region of defects stained with Masson-Goldner trichrome staining. The yellow dotted boxes indicate the defined region of interest (ROI) (3.0 mm × 4.0 mm) selected for the histomorphometric analyses. The ROI was divided into superficial, middle and deep zone (each zone 1.0 mm × 4.0 mm). (**b**) No significant differences were seen between the three groups for BV/TV, Tb.Th, Tb.Sp, and Tb.N. (**c**) Typical higher-magnification image of the interface between the repair tissue and the subchondral bone, showing an accumulation of osteoclasts (black arrowheads) and osteoblasts (black arrows) as well as newly formed bone (black stars). The asterisks indicate the marrow cavity and the white arrowheads indicate blood vessels. (**d**) Density of osteoclasts and osteoblasts within the defined ROI. Compared with enhanced microfracture and debridement only, a significant higher density of osteoclasts was detected in the microfracture group (without bone marrow aspirate). Microfracture without and with bone marrow aspirate recruited more osteoblasts than debridement alone to the treated subchondral bone. (**e**) Percentage of areas occupied by marrow cavity (white), original (mature) bone (dark red), new bone (light red), and repair tissue (green) within the defined ROI of defects from the three individual groups. Compared with microfracture alone, a significant larger area of marrow cavity and higher preservation rate of original (mature) bone were observed in the enhanced microfracture group. *Indicates a significant difference between debridement and microfracture group, ^#^indicates a significant difference between debridement and enhanced microfracture, and ^§^indicates difference between microfracture and enhanced microfracture. Scale bars: 1.0 mm (**a**); 200 μm (**c**).

Histomorphometric analysis within the defined region of interest (ROI) of the subchondral bone below the cartilage defects revealed no statistically significant differences of bone volume fraction (BV/TV), mean trabecular thickness (Tb.Th), mean trabecular separation (Tb.Sp), and mean trabecular number per length unit (Tb.N) between the three groups (all *P* > 0.05) (Fig. 3a,b; Table 1). Microfracture alone elicited a significantly higher density of osteoclasts than enhanced microfracture (microfracture: 41.50 ± 19.14; enhanced microfracture: 18.17 ± 4.79; *P* = 0.012) (Fig. 3c,d; Table 1). Osteoclasts were evenly distributed within the superficial, middle and deep zones of the entire ROI (all zones: *P* < 0.05 for microfracture alone *versus* debridement and enhanced microfracture). Total osteoblast density was not different between the 3 groups (*P* > 0.05). Interestingly, in the middle zone of the ROI, both microfracture without and with aspirate recruited significantly more osteoblasts when compared with debridement alone (3.1- and 2.6-fold, respectively; both *P* < 0.05 for debridement *versus* microfracture without and with aspirate). Microfracture alone significantly reduced the amount of mature bone compared with debridement only (*P* = 0.013; Fig. 3e). Addition of bone marrow aspirate resulted in a significantly reduced new bone formation compared to microfracture alone (*P* = 0.008; Fig. 3e), while it did not significantly affect the amount of mature bone (*P* > 0.05).

#### Qualitative evaluation of subchhondral bone changes within the defect regions

**Figure 4:**
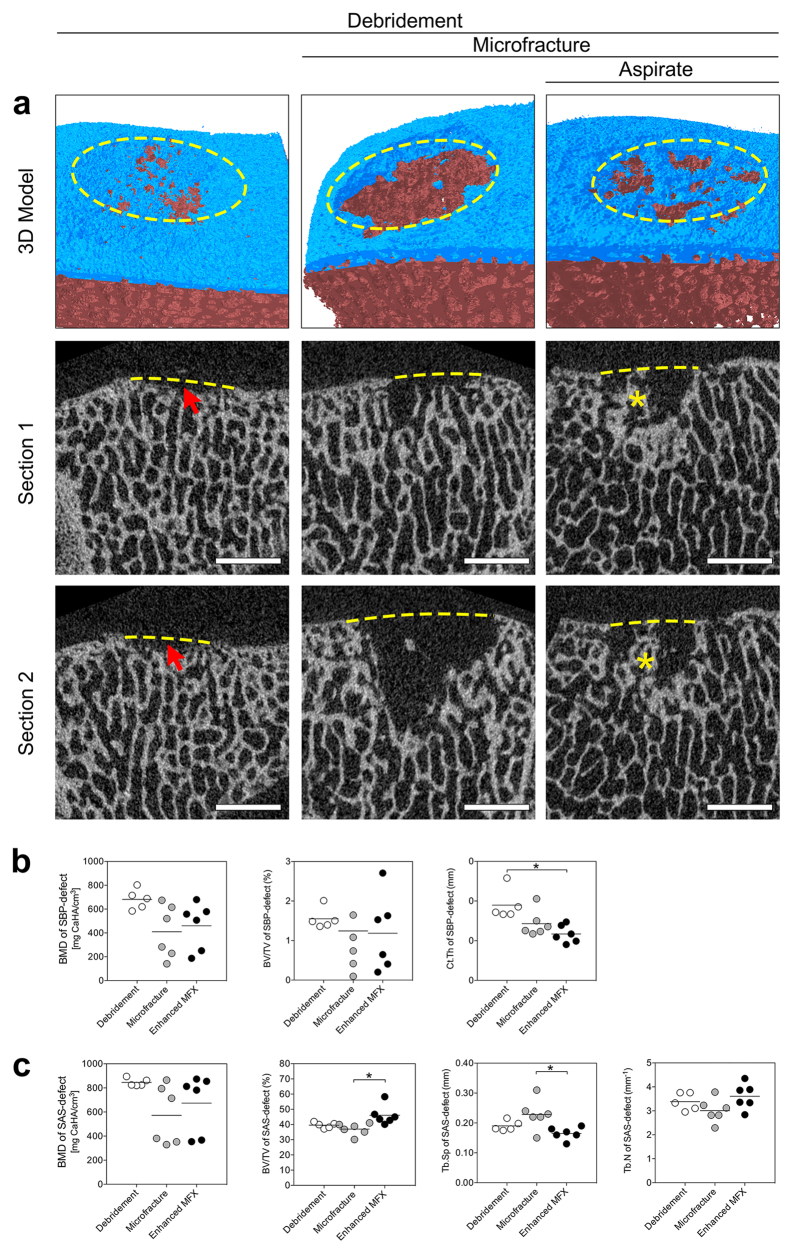
Qualitative and quantitative analysis of subchondral bone changes underlying defects treated with the three strategies. (**a**) Representative micro-CT images of subchondral bone changes. The top row shows a 3D reconstruction of characteristic defects. The subchondral bone plate and subarticular spongiosa are colored in blue and red, respectively. The yellow dashed ellipse shows the margin of the defect. The middle and bottom rows represent two different sections of the defects of the top row. Removal of the calcified cartilage layer in the debridement group induced subchondral bone loss (red arrows). In defects from the microfracture and enhanced microfracture group, residual microfracture holes and peri-hole bone resorption were present. The yellow dashed lines indicate the projected cement lines, and the red asterisks indicate the preserved bone bridge between two residual microfracture holes. Scale bar: 2 mm. (**b**) Comparison of the micro-CT parameters of SBP-defect between the three treatment groups. ^*^P < 0.05. (**c**) Comparison of the micro-CT parameters of SAS-defect between the three treatment groups. ^*^P < 0.05.

According to a micro-CT based algorithm for the analysis of subchondral bone changes following microfracture treatment^25^, no intra-lesional osteophytes and subchondral bone cysts were detected in defects from any treatment group. Residual microfracture holes and peri-hole bone resorption were frequently observed (Fig. 4a; Table 2).

In defects treated with microfracture, only 2 of 18 holes (11%) were preserved as residual microfracture holes, while peri-hole bone resorption was observed in 16 of 18 holes (89%). In defects treated with enhanced microfracture, the incidence of residual microfracture holes was 6/18 (33%), and peri-hole bone resorption was 12/18 (67%). Yet, no significant differences in maximal horizontal diameter, vertical diameter, area, and bone bridge height were detected between the microfracture and enhanced microfracture group.

#### Quantitative analysis of subchondral bone changes in the defect and adjacent regions

The subchondral bone plate below the defects attained a significant decrease of bone volume fraction (BV/TV), specific bone surface (BS/BV), and bone surface density (BS/TV) when compared with the adjacent subchondral bone plate, irrespective of the treatment strategy (Supplementary Table S4). Besides, both microfracture and enhanced microfracture treatments yielded a significant reduction in bone mineral density (BMD) of the subchondral bone plate beneath the defects when compared with the adjacent subchondral bone plate. No differences in cortical thickness of defect *versus* adjacent subchondral bone plate were found between all groups.

In the subarticular spongiosa below the articular cartilage defects, debridement yielded a reduced bone volume fraction, trabecular thickness (Tb.Th) and structure model index (SMI) when compared with the adjacent subarticular spongiosa. Microfracture alone induced a general reduction of bone surface density (BS/TV), trabecular number (Tb.N), and degree of anisotropy (DA) as well as an increased trabecular separation (Tb.Sp) compared with the adjacent spongiosa. In defects treated with enhanced microfracture, a decrease of specific bone surface, trabecular pattern factor (Tb.Pf), structure model index, and degree of anisotropy was observed in the subarticular spongiosa of the defect region (Supplementary Table S4).

#### Quantitative analysis of subchondral bone changes between the three different treatment groups

At the level of subchondral bone plate, debridement alone led to less severe osseous deterioration when compared with the microfracture and enhanced microfracture group as indicated by the highest volume of cortical thickness (debridement: 0.10 mm ± 0.02 mm; microfracture: 0.07 mm ± 0.02 mm; enhanced microfracture: 0.06 mm ± 0.01 mm; *P* = 0.006) (Fig. 4b; Table 3). Values for bone mineral density were also more preserved with debridement alone without reaching significance (debridement: 630.79 mg CaHA/cm^3^ ± 85.34 mg CaHA/cm^3^; microfracture: 410.05 mg CaHA/cm^3^ ± 222.01 mg CaHA/cm^3^; enhanced microfracture: 460.05 mg CaHA/cm^3^ ± 196.34 mg CaHA/cm^3^; *P* = 0.066).

At the level of subarticular spongiosa (SAS-defect), an increased bone volume fraction (46.07% ± 6.38%) resulted in the bone marrow aspirate enhanced microfracture group when compared with both microfracture alone (36.91% ± 3.90%; *P* = 0.008; 0.8-fold) and debridement only (40.08% ± 2.13%; *P* = 0.085; 0.9-fold). Enhanced microfracture with aspirate also led to a significantly lower trabecular separation (microfracture: 0.23 mm ± 0.05 mm; enhanced microfracture: 0.17 mm ± 0.02 mm *P* = 0.015), a higher trabecular number (microfracture: 3.01 ± 0.50; enhanced microfracture: 3.61 ± 0.53*P* = 0.108), and a reduced degree of anisotropy (microfracture: 0.44 ± 0.03; enhanced microfracture: 0.39 ± 0.03*P* = 0.088) when compared with microfracture alone (Fig. 4c; Table 3). A comparison between debridement and microfracture group revealed no statistically significant differences within the subarticular spongiosa (0.093 < *P* < 0.988) (Fig. 4c; Table 3).

#### Mathematical modeling of subchondral bone volume changes within defect regions

**Figure 5:**
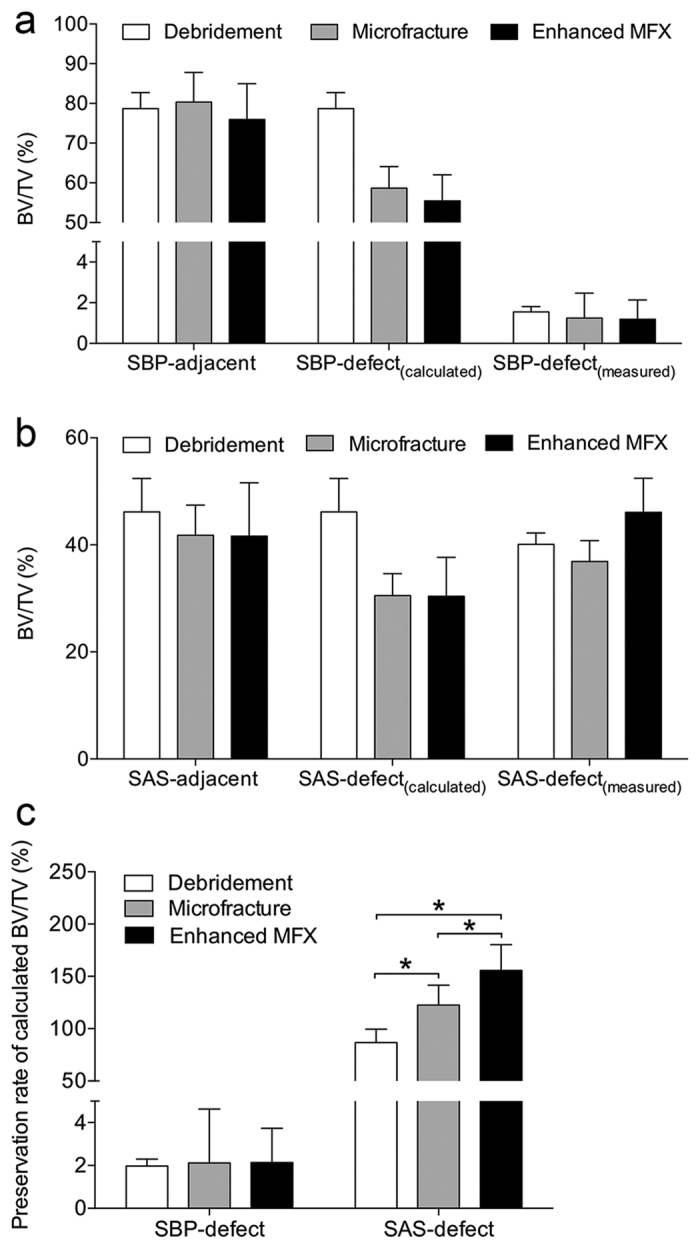
Mathematical modeling of subchondral bone volume changes within defect region following the three treatments. (**a**) Comparison of the calculated BV/TV of SBP-defect [SBP-defect(calculated)] and the measured BV/TV of SBP-defect [SBP-defect(measured)]. (**b**) Comparison of BV/TV of SAS-defect(calculated) and BV/TV of SAS-defect(measured). (**c**) Comparison of preservation rate of BV/TV of SBP-defect(calculated) and SAS-defect(calculated) of the three groups. No apparent differences were found in preservation rates of BV/TV of SBP-defect(calculated) between the three groups, but additional application of aspirates yielded the highest preservation rate of BV/TV of SAS-defect(calculated). *P < 0.05.

A mathematical comparison between the measured and calculated values of bone volume fraction of the treated subchondral bone plate showed a similar bone preservation rate in the three treatment groups (debridement: 1.97% ± 0.33%; microfracture: 2.12% ± 2.51%; enhanced microfracture: 2.14% ± 1.59%; *P* = 0.966) (Fig. 5a,c; Supplementary Table S5). Within the subarticular spongiosa of the defect region, in contrast, compared with the other two groups, the enhanced microfracture yielded the significantly highest preservation rate of bone volume fraction (debridement: 85.69% ± 12.78%; microfracture: 122.54% ± 18.98; enhanced microfracture: 155.72% ± 24.51%; *P* = 0.0002; 1.8-fold compared with debridement) (Fig. 5b,c; Supplementary Table S6).

## Discussion

In the present study we tested the hypothesis that early osteochondral repair following microfracture is enhanced if bone marrow aspirate is added as an adjunct to the microfracture procedure. The key finding is that microfracture treatment of chondral defects induced a substantial early loss of the subchondral bone that may be significantly counteracted if bone marrow aspirate is applied. Second, such bone marrow aspirate enhanced microfracture also significantly protected the subarticular spongiosa below the defects from osteoclast-mediated peri-hole bone resorption compared with microfracture alone. Interestingly, debridement down to the subchondral bone similarly led to a reduction of the bone volume both in the subchondral bone plate and subarticular spongiosa. Finally, debridement and microfracture without or with bone marrow aspirate were all equivalent regarding the early phase of articular cartilage repair.

Microfracture treatment significantly reduced the amount of mature bone compared with debridement only, as determined by histomorphometrical analysis. The additional application of bone marrow aspirate to microfracture led, in the subarticular spongiosa, to a significantly higher bone volume fraction and increased trabecular number, together with a decreased trabecular separation compared with microfracture alone as determined by micro-CT analysis. In good agreement, the bone marrow aspirate treatment restrained the reaction of osteoclasts within the subchondral bone, thus acting towards preserving the original subchondral bone (microfracture: 25.9% *versus* enhanced microfracture: 31.3%). Together with the data from the histomorphometrical analyses, these findings suggest that microfracture treatment induces a substantial early loss of the subchondral bone that may be significantly counteracted by bone marrow aspirate, which however does not seem to primarily act via a stimulation of new bone formation in the region below the defects. It is possible, however, that such a stimulation of new bone formation may be seen at later time points. Moreover, the additional marrow aspirate yielded a change towards presence of more residual microfracture holes (microfracture: 11% *versus* enhanced microfracture: 33%) *vis-à-vis* less peri-hole bone resorption (microfracture: 89% *versus* enhanced microfracture: 67%). Besides, the preservation rate of bone volume fraction of the subarticular spongiosa of defect region following microfracture was also significantly improved by addition of bone marrow aspirate. These data suggest a protective effect of the bone marrow aspirate on the subchondral bone, especially the subarticular spongiosa, which is also consistent with previous studies on the osteogenic effect of bone marrow aspirate in fracture and osteonecrosis treatments^9^,^26^,^27^. It is possible that the bone marrow aspirate protected the openings of the microfracture holes towards the joint space from the aggressive synovial fluid, thereby additionally favoring new tissue formation^4^,^8^,^28^.

The present study also demonstrated that debridement alone significantly affected the subchondral bone, which per definition was not perforated by the sole removal of the calcified cartilage layer. Of note, the great amount of bone loss occurred within the entire subchondral bone of the defect region in the debridement group, and the extent of bone loss of the subchondral bone plate of defect region was similar to that in the other two groups. However, the histological subchondral bone evaluation using the Sellers score showed an improved value in the debridement group compared with the microfracture treatment. Besides, the micro-CT calculation revealed that debridement retained the broadest subchondral bone plate among the three groups.

Regarding short-term outcomes of cartilage repair, debridement and microfracture functioned equivalently in this model. This raises the question whether the additional penetration of the subchondral bone plate might be unnecessary for the generation of a fibrocartilage-like repair tissue. After meticulous debridement, i.e. removal of the calcified cartilage layer, bleeding from the subchondral bone plate is often observed immediately within the defect in patients^29^. Such bleeding mostly originates from the opened vessels of the subchondral bone extending upward into the overlying calcified cartilage layer^13^. The subsequently formed so-called “super clot” consists of bone marrow extracellular matrix, progenitor cells, and stem cells, which may be sufficient for the repair of the lesion^30^,^31^. Possibly, this “super clot” is capable of fulfilling the same effects than the additional bone marrow aspirate in a clinical setting.

Interestingly, addition of bone marrow aspirate to the microfracture treatment did not further improve cartilage repair compared with microfracture alone. One possible explanation for this finding is the relative paucity of the progenitor cells in the native, non-centrifuged bone marrow aspirate^32^,^33^. However, some authors questioned the difference of the cellular components between native and centrifuged marrow aspirate^26^,^34^. The native bone marrow aspirate was even proven to be superior to centrifuged bone marrow aspirate for the healing of meniscal lesions in a dog model^35^. Another possible explanation is the short observation period in the present study. In previous studies with encouraging results of native or centrifuged bone marrow aspirate, evaluations were performed at 3 months^35^, 8 months^4^, and even 10 years^32^.

Enhanced microfracture techniques aim to improve standard microfracture by using matrices to stabilize the mesenchymal clot following microfracture treatment and to improve mesenchymal stem cell differentiation into chondrocytes. These procedures chiefly include autologous matrix-induced chondrogenesis (AMIC)^36^ and commercially available enhanced marrow-stimulation products^37^,^38^,^39^. However, reports of clinical outcomes for patients treated with enhanced microfracture technique for articular cartilage repair remain limited to date, and long-term outcomes are unavailable. Although most available literature of biomaterials enhanced microfracture do report promising clinical results in case series^40^,^41^,^42^, further preclinical and randomized, double-blinded controlled clinical studies with larger cohorts and longer follow-up are required before any conclusions can be drawn regarding the short- and long-term effectiveness of these techniques.

From a clinical standpoint, the present study and previous investigations in small animal models^19^,^22^,^43^ show that the balance of bone turnover is clearly shifted to bone resorption at this very early phase, the first several weeks of cartilage repair following microfracture. The finding of the present study that osteoclast density is higher in both microfracture groups compared with debridement alone is in good agreement with the work of Chen *et al*., supporting the important role of osteoclasts as cellular mediators of marrow stimulation techniques that incise the subchondral bone^22^. Remarkably, previous clinical studies reported pathological alterations of the subchondral bone at later time points. Especially subchondral bone cysts and intralesional osteophytes were seen in up to one third of patients treated with microfracture as early as 6 months postoperatively^18^,^44^,^45^. Together with the upward migration of the subchondral bone plate, these phenomena possibly play a role in the degeneration of the cartilaginous repair tissue over time^16^,^46^,^47^,^48^. As they weaken the subchondral bone support of the cartilaginous repair tissue, these data highlight the importance of the clinical treatment regimen of a protected weight bearing for the first 6 weeks postoperatively, as recommended for all marrow stimulation techniques in the tibiofemoral compartment of the human knee joint. Moreover, these data also serve to instigate more clinical observations of the early postoperative phase, e.g. by using MRI or cone beam CT^49^ to search for similar changes of the subchondral bone in patients.

This study holds some limitations. Animals were allowed immediate full weight-bearing and received no postoperative rehabilitation regimes, which might alter the outcome of the postoperative repair of the osteochondral unit compared with the clinical situation. Also, only one single cartilage defect was created per knee in the debridement group, while two defects per knee were generated in the microfracture or enhanced microfracture group. Yet, Christensen *et al*.^50^ observed no difference of cartilage repair between multiple or singular defects per knee. Finally, the 4-week time point selected here to study early osteochondral repair might be not long enough for an overall and final assessment. Strengths of this investigation include the use of a translational animal model and the choice of an early time point, allowing for an in-depth analysis of the early effects of marrow stimulation on the osteochondral unit with a broad variety of robust evaluation methods.

In summary, the data show that microfracture treatment induced a significant early loss of the subchondral bone. Moreover, the data suggest that the additional application of bone marrow to defects may counteract such loss of subchondral bone in this translational model of osteochondral repair. Compared with isolated removal of the calcified cartilage layer (debridement), penetration of the subchondral bone plate (microfracture) was more deleterious for the osteochondral unit than previously expected. This finding has several important clinical implications. One is to carefully reconsider the very roles of debridement and microfracture for articular cartilage repair. Furthermore, the data indicate a potential bone protective capacity of the marrow aspirate, supporting its application accompanying other procedures. Clinical studies are warranted to further elucidate both early events of osteochondral repair and the effect of such adjunct treatments.

## Methods

### Study design

Standardized circular (diameter 4 mm) full-thickness chondral defects in the trochlear groove of minipigs were treated by (1) debridement (debridement group), (2) debridement and microfracture (microfracture group), and (3) debridement, microfracture, and bone marrow aspirate (enhanced microfracture group) (Fig. 1). In the debridement group, the entire calcified cartilage layer was meticulously debrided down to the subchondral bone plate. In the microfracture group, following an identical debridement, three uniform microfracture holes with standardized diameter of 1.2 mm and depth of 5.0 mm were introduced. Here, a custom-made microfracture awl with a straight trihedral cutting tip and a penetration stop was used. Defects of the enhanced microfracture group were treated as described above with an addition of a layer of fresh autologous bone marrow aspirate, which was retrieved from the ipsilateral proximal tibia. Osteochondral repair was assessed at 4 weeks postoperatively using established macroscopic, histological, immunohistochemical, and micro-computed tomography analyses^51^. The cartilage and subchondral bone adjacent to the treated osteochondral region served as internal controls.

### Animal experiments

All animal experiments were conducted in agreement with the national legislation on protection of animals and the National Institutes of Health (NIH) Guidelines for the Care and Use of Laboratory Animals (NIH Publication 85–23, Rev 1985) and were approved by the Saarland University Animal Committee according to German guidelines.

In the defect preparation and treatment surgery, 11 skeletally mature, healthy female Göttingen minipigs (age between 18 and 22 months; body weight (BW) 38.9 ± 5.3 kg) received water *ad libitum* and were fed a standard diet. A veterinarian continuously monitored all animals. Following a 12-hour fast, the animals were sedated with intramuscular injection of 30 mg ketamine/animal (Ketanest S, Pfizer, Berlin, Germany), 2 mg xylazine/animal (Rompun, Bayer, Leverkusen, Germany), and 1 mg atropine/animal (B. Braun, Melsungen, Germany), and endotracheally intubated after intravenous administration of 20 ml of 2% propofol (AstraZeneca, Wedel, Germany). General anesthesia was maintained by inhalation of 1.5% isoflurane (Baxter, Unterschleißheim, Germany) and intravenous administration of propofol (6–20 mg/kg BW/h). Unilateral surgery was performed in the hind legs with right and left knees alternating within groups. The surgical approach was performed as described elsewhere^50^. The minimal sample size calculation was performed as previously described^52^. In 5 minipigs, 1 defect per animal were created and treated with debridement alone. In another 6 minipigs, 2 defects per animal were created ipsilaterally, and treated with microfracture or bone marrow aspirate enhanced microfracture, respectively.

Standardized circular (diameter 4 mm) full-thickness chondral defects (n = 17) were lined on the lateral facets of the femoral trochlea with a custom-made skin punch (Kai Europe, Solingen, Germany). In the debridement group (n = 5), defects only received the debridement treatment. For the debridement, the entire calcified cartilage layer was meticulously debrided down to the subchondral bone plate which was left intact and did not receive any abrasion using a ring curette (Aesculap, Tuttlingen, Germany) (Fig. 1a). In the microfracture group (n = 6), following identical debridement, three uniform microfracture holes (diameter 1.2 mm, depth 5.0 mm) were then introduced in a standardized manner, perpendicular to the joint surface and evenly distributed within the defect. A custom-made microfracture awl with a straight trihedral cutting tip and a penetration stop was used to standardize the penetration depth to 5.0 mm (Aesculap, Tuttlingen, Germany) (Fig. 1c). In the enhanced microfracture group (n = 6), following debridement and microfracture, an additional layer of fresh autologous bone marrow aspirate retrieved from the ipsilateral proximal tibia was injected onto the bottom of the defects treated with microfracture (25 μl per defect) (Fig. 1a,b). Joints were closed in layers. The animals were allowed immediate full weight-bearing. Fentanyl pain patch with a release rate of 100 μg/h was applied for 72 hours postoperatively. If needed, 4 mg/kg BW caprofen (Rimadyl, Pfizer) were admitted orally during the postoperative period.

Animals were sacrificed in general anesthesia at 4 weeks postoperatively. Digital photographs of the entire defect area (n = 17) were obtained under standardized illumination conditions^24^ at the time of animal sacrifice to allow for macroscopic evaluation. Osteochondral specimens containing the cartilage defects were dissected in a standardized manner, placed in 4% formalin for 24 hours and stored in 70% ethanol thereafter. Following micro-CT analysis, specimens were decalcified with 5% formic acid, trimmed, and proceeded for histological and immunohistological evaluations.

### Evaluation of articular cartilage repair

Photographs for the macroscopic grading of articular cartilage repair were independently evaluated by two blinded experienced investigators using the inverse scoring system by Goebel *et al*.^23^ (20 = no repair; 0 = normal articular cartilage).

For the histological evaluation, sections (4 μm) were obtained using a microtome cutting from the center of each paraffin-embedded specimen and stained with safranin orange/fast green (safranin O) and hematoxylin and eosin (HE) as previously described^53^,^54^. A total of 136 stained sections (8 sections per defect) were analyzed by two independent investigators applying the complex cartilage repair score described by Sellers *et al*.^24^. Values ranged from 31 (empty defect without repair tissue) to 0 points (complete regeneration).

For the evaluation of immunoreactivity to type-II collagen, the paraffin-embedded sections were submerged in 0.3% hydrogen peroxide for 30 min. After washing with PBS, sections were incubated for 30 minutes in 0.1% trypsin, washed with PBS and blocked with 3% bovine serum albumin in PBS (blocking buffer) for 30 minutes. The sections were then incubated with a 1/50 dilution of a monoclonal mouse anti-human type-II collagen IgG (Acris, Hiddenhausen, Germany) in blocking buffer for 24 hours at 4 °C, washed and exposed to a 1/100 dilution of a biotinylated anti-mouse antibody (Vector Laboratories, Burlingame, CA) for 1 hour at room temperature. The sections were washed, incubated for 30 minutes with avidin-biotin-peroxidase reagent, and then exposed to diaminobenzidine. Immunoreactivity to type-II collagen in the repair tissue was compared with that of the adjacent articular cartilage, serving as positive internal control, and reported as a semiquantitative grading (0: no immunoreactivity; 1: significantly weaker; 2: moderately weaker; 3: similar; 4: stronger immunoreactivity)^51^.

All microscopic images were acquired by a solid-state CCD camera mounted on a microscope (BX-45; Olympus, Hamburg, Germany) and analyzed with the analySIS software (Soft Imaging System GmbH, Münster, Germany) using standardized parameters.

### Evaluation of subchondral bone reconstitution

#### Histomorphometric analysis of the subchondral bone status

Paraffin embedded sections were processed for Masson-Goldner trichrome staining as previously described^55^. A standardized region of interest (ROI) was set up covering the trabecular region of the subchondral bone underlying the treated defects with 3.0 mm depth and 4.0 mm width (Fig. 3a). Quantitative histomorphometry was performed in sections through the central region of defects using Map_Bonemicrostructure, a plugin for ImageJ 1.50 (National Institutes Health, MD, USA)^56^, and Adobe Photoshop CC 2015 (Adobe Systems, Mountain View, CA, USA)^57^. Map_BoneMicrostructure calculated the following standard bone histomorphometry parameters on a digitized trabecular bone surface according to the parallel plates model^58^: bone volume fraction (BV/TV; %), mean trabecular thickness (Tb.Th; μm), mean trabecular separation (Tb.Sp; μm), and mean trabecular number per length unit (Tb.N; 1/mm). Osteoclast and osteoblast density was determined and quantified by manual point counting within 3 zones (each zone 1.0 mm × 4.0 mm) of the entire ROI using the following criteria^59^ (Fig. 3a). Criteria for the osteoclast identification involved: (1) multinuclear cell (≥3 nuclei), (2) concave surface of bone beside the cell, and (3) larger cytoplasm than nucleus. Criteria for the osteoblast identification included: (1) cuboidal or polygonal cell without concave surface of bone beside cell and (2) space between cells and mature bone which was filled by osteoid. The osteoid (new formed bone) was lined with osteoblasts and prominently vascularized between cells and mineralized mature bone. For each defect, the percentage of marrow cavity, original (mature) bone, new bone (osteoid), and repair tissue was quantified within two Masson-Goldner trichrome stained sections per defect through the central region of the defect using the ImageJ software. The nomenclature, symbols, and units complied with the report of the American Society for Bone and Mineral Research Nomenclature Committee^60^.

#### Qualitative evaluation of subchondral bone changes

The subchondral bone plate and the subarticular spongiosa beneath cartilage defects and laterally adjacent to the defects (internal controls) were separately assessed as previously described^61^ using a microfocused X-ray computed tomography (micro-CT) scanner (Skyscan 1172; Bruker, Belgium) with a maximal nominal resolution below 0.8 μm.

**Figure 6:**
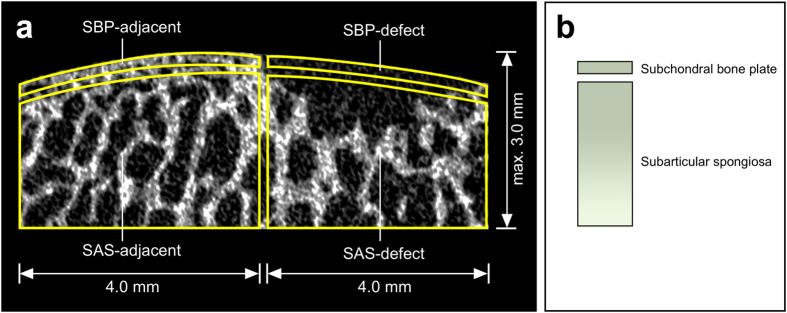
Standardized regions of interest (ROI) for the quantitative micro-CT evaluation of the subchondral bone. (**a**) Four standardized ROIs were defined on micro-CT images. SBP-defect involved exclusively the subchondral bone plate within the defect, and the underlying subarticular spongiosa (SAS-defect). SBP-adjacent and SAS-adjacent were located laterally neighboring SBP-defect and SAS-defect correspondingly. The maximum total vertical depth of SBP and SAS was 3.0 mm. Overlapping of individual ROIs was strictly avoided. (**b**) Schematic of the ROIs for subchondral bone evaluation. Note the ~10-fold larger ROI of the subarticular spongiosa compared with the subchondral bone plate.

For each osteochondral specimen, four distinct volumes of interest (VOIs) were evaluated within the subchondral bone compartment using the CTAnalyzer software (Bruker Skyscan, Kontich, Belgium)^61^, including “subchondral bone plate-defect (SBP-defect)”, “subarticular spongiosa-defect (SAS-defect)”, “subchondral bone plate-lateral adjacent (SBP-adjacent)”, and “subarticular spongiosa-lateral adjacent (SAS-adjacent)” (Fig. 6). SAS-adjacent was placed peripherally on the femoral trochlea. The total vertical depth of all VOIs was limited to 3.0 mm, and the maximal horizontal diameter of each VOI was restricted to 4.0 mm. Overlapping of VOIs was avoided.

Based on the micro-CT image dataset of each osteochondral specimen, the presence of subchondral bone alterations was described and qualitatively evaluated^25^. The reported types of subchondral bone changes included intra-lesional osteophytes, residual microfracture holes, peri-hole bone resorption, and subchondral bone cysts. For intra-lesional osteophytes, the maximal height, maximal width, maximal two-dimensional (2D) area, and relative location of each osteophyte were reported. For residual microfracture holes, peri-hole bone resorption, and subchondral bone cysts, the maximal height of bone bridges (if existing), maximal horizontal diameter, maximal vertical diameter, and maximal 2D area were determined as previously described.

#### Quantitative evaluation of subchondral bone changes

For the quantitative analysis of the subchondral bone compartment, the following variables were determined within each of the four VOIs in a three-dimensional (3D) fashion^61^: bone mineral density (BMD), bone volume fraction (BV/TV), specific bone surface (BS/BV), bone surface density (BS/TV). Cortical thickness (Ct.Th) was only reported within the subchondral bone plate, while trabecular thickness (Tb.Th), trabecular separation (Tb.Sp), trabecular pattern factor (Tb.Pf), trabecular number (Tb.N), structure model index (SMI), degree of anisotropy (DA), and fractal dimension (FD) were only determined in the subarticular spongiosa.

#### Mathematical modeling of subchondral bone volume changes within the defect region

In each group, the real values of bone volume fraction (BV/TV) of SBP-defect and SAS-defect were directly measured by the CTAnalyzer software and referred to as SBP-defect_(measured)_ and SAS-defect_(measured)_, respectively.

In the debridement group, the entire subchondral bone was preserved theoretically (Fig. 1a,b). Therefore, using adjacent VOIs as references, the expected value of BV/TV of SBP-defect [SBP-defect_(calculated)_] and SAS-defect [SAS-defect_(expected)_] were determined as:


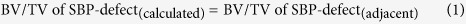



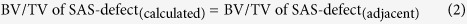


**Figure 7:**
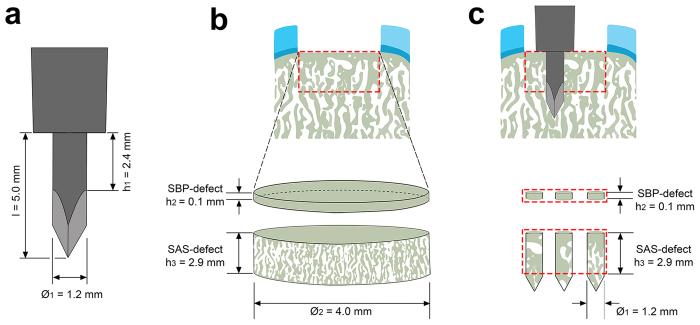
Mathematical modeling for calculating the change of subchondral bone volume following the microfracture procedure. (**a**) Schematic of the microfracture awl tip of Fig. 4c.(**b**) Volume of interest (VOI) of the entire subchondral bone underlying the full-thickness chondral defect is composed of two cylinders. The top cylinder is VOI of SBP-defect with height 0.1 mm (average value of 20 measurements) and diameter 4.0 mm, and the bottom one is VOI of SAS-defect with height 2.9 mm and diameter 4.0 mm. (**c**) In this model, the three microfracture impactions excise triple volume of the microfracture awl tip. Of note, when interacting with the subchondral bone, most of the trihedral head of the awl tip penetrates beyond the defined region of VOI of the subchondral bone (SBP-defect and SAS-defect). Therefore, the excised subchondral bone within the VOI of the subchondral bone is approximately equal to two cylindrical sections: one is within SBP-defect (height 0.1 mm; diameter 1.2 mm), and the other is within SAS-defect (height 2.9 mm; diameter 1.2 mm).

In the microfracture and enhanced microfracture group, the interaction between the microfracture awl and the subchondral bone was modeled (Fig. 7). Here, the awl tip was composed of a distal trihedral head (length 2.6 mm), a middle cylindrical body (length 2.4 mm), and a distal penetration stop, standardizing the depth of penetration to 5.0 mm (Fig. 7a). As the maximal vertical depth of VOIs of SBP-defect and SAS-defect was defined within 3.0 mm (Figs 6a and 7b), and the thickness of SBP-defect was 0.1 mm (average value after 20 measurements), the volume occupied by the awl tip within the defined VOIs (SBP-defect and SAS-defect) approximated a cylindrical configuration with a height of 3.0 mm and a base diameter of 1.2 mm (Fig. 7c). Therefore, BV/TV of SBP-defect_(calculated)_ and SAS-defect_(calculated)_ were calculated as follows:


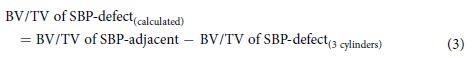



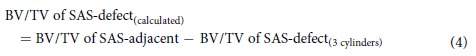


### Statistical Analysis

Values are expressed as mean ± standard deviation (SD). One-way ANOVA with Tukey’s post-hoc test and Mann-Whitney U test were used where appropriate. Any *P* value < 0.05 was considered statistically significant. Calculations were performed using SPSS (IBM SPSS 20; SPSS Inc., Chicago, IL).

## Additional Information

**How to cite this article:** Gao, L. *et al*. Early loss of subchondral bone following microfracture is counteracted by bone marrow aspirate in a translational model of osteochondral repair. *Sci. Rep.* **7**, 45189; doi: 10.1038/srep45189 (2017).

**Publisher's note:** Springer Nature remains neutral with regard to jurisdictional claims in published maps and institutional affiliations.

## Supplementary Material

Supplementary Materials

## Figures and Tables

**Table 1:** Histomorphometric analysis of the subchondral bone below the treated cartilage defects.

Parameter	Unit	Debridement	Microfracture	Enhanced microfracture	Overall *P*	Specific *P*		
						*	#	§
Bone structure								
BV/TV	%	48.06 ± 10.23	46.8 ± 13.45	56.13 ± 12.69	0.395	0.984	0.541	0.413
Tb.Th	mm	233.20 ± 49.51	255.88 ± 69.96	289.62 ± 107.64	0.523	0.890	0.502	0.756
Tb.Sp	mm	254.06 ± 65.52	295.53 ± 87.44	215.15 ± 65.03	0.206	0.633	0.668	0.180
Tb.N	mm^−1^	2.10 ± 0.23	1.83 ± 0.23	2.13 ± 0.73	0.514	0.635	0.993	0.536
Osteoclast density								
Superficial zone	−/−	8.33 ± 3.27	16.80 ± 6.53	7.33 ± 2.50	**0.005**	**0.014**	0.914	**0.007**
Middle zone	−/−	4.67 ± 3.14	15.00 ± 4.12	6.00 ± 1.90	**0.001**	**0.001**	0.743	**0.001**
Deep zone	−/−	5.83 ± 3.43	16.80 ± 6.53	4.83 ± 3.76	**0.015**	**0.034**	0.937	**0.018**
Total	−/−	19.00 ± 4.30	41.50 ± 19.14	18.17 ± 4.79	**0.008**	**0.020**	0.993	**0.012**
Osteoblast density								
Superficial zone	−/−	117.67 ± 65.89	152.80 ± 82.89	176.50 ± 101.90	0.501	0.777	0.473	0.891
Middle zone	−/−	65.67 ± 45.20	204.00 ± 72.49	172.33 ± 69.80	**0.006**	**0.007**	**0.028**	0.691
Deep zone	−/−	86.50 ± 64.24	152.80 ± 82.89	181.33 ± 118.11	0.119	0.147	0.201	0.958
Total	−/−	271.20 ± 104.53	505.00 ± 212.31	513.50 ± 246.38	0.123	0.173	0.154	0.997
Area within ROI								
Marrow cavity	mm^2^	6.21 ± 1.23	3.47 ± 0.68	5.16 ± 0.50	**0.004**	**0.004**	0.173	**0.034**
Mature bone	mm^2^	4.33 ± 0.84	3.11 ± 0.17	3.75 ± 0.12	**0.016**	**0.013**	0.197	0.145
New bone	mm^2^	0.69 ± 0.25	1.37 ± 0.47	0.54 ± 0.06	**0.007**	**0.021**	0.679	**0.008**
Repair tissue	mm^2^	0.78 ± 0.14	4.05 ± 0.04	2.55 ± 0.33	**0.001**	**0.001**	**0.001**	**0.001**

Values are expressed as mean ± SD. Bold values indicate a significant difference between groups (*P* < 0.05). No differences of bone volume fraction (BV/TV), mean trabecular thickness (Tb.Th), mean trabecular separation (Tb.Sp), and mean trabecular number per length unit (Tb.N) were detected with histomorphometry of the defined region of interest within the underlying subchondral bone of defects from the three treatment groups. Of note, a significant increase of osteoclast density was identified in defects treated with microfracture alone compared with bone marrow aspirate enhanced microfracture. Osteoblast density was higher in both the microfracture alone and enhanced microfracture group compared with debridement only. Also, the area of mature bone was significantly more preserved when bone marrow aspirate was applied to the microfracture procedure compared with microfracture alone, suggesting a positive inhibitory effect on the bone resorption resulting from the microfracture procedure. Overall *P* values were for comparisons among the three treatment groups. Specific *P* values were for comparisons of two of the three treatment groups as follows: ^*^P < 0.05 for debridement *versus* microfracture; ^#^*P* < 0.05 for debridement *versus* enhanced microfracture; ^§^*P* < 0.05 for microfracture *versus* enhanced microfracture.

**Table 2:** Comparative and descriptive results of the subchondral bone analysis for residual microfracture hole and peri-hole bone resorption on micro-computed tomography.

	Microfracture	Enhanced microfracture	*P* values
	(n = 18 holes)	(n = 18 holes)	
Residual microfracture holes			
Number (%)	2 (11%)	6 (33%)	0.228
Horizontal diameter (mm)	1.70 ± 0.02	1.50 ± 0.22	0.290
Vertical diameter (mm)	2.07 ± 0.13	2.43 ± 0.53	0.290
Area (mm^2^)	1.91 ± 0.60	2.02 ± 0.36	0.640
Bone bridge height (mm)	2.16 ± 0.51	1.97 ± 0.83	0.860
Peri-hole bone resorption			
Number (%)	16 (89%)	12 (67%)	0.228
Horizontal diameter (mm)	3.29 ± 0.22	3.43 ± 0.19	0.570
Vertical diameter (mm)	2.29 ± 0.67	2.16 ± 1.19	1.000
Area (mm^2^)	4.18 ± 1.96	2.73 ± 1.02	0.390

Both the microfracture and enhanced microfracture group contained 6 cartilage defects per group (one per animal); each defect always received 3 microfracture penetrations (holes). Values are reported as mean ± standard error of the mean unless otherwise indicated. More residual microfracture holes and less peri-hole bone resorption were detected in the enhanced microfracture group. No significant difference existed for all parameters between the microfracture and enhanced microfracture group (all *P *> 0.05).

**Table 3:** Comparison of the micro-CT parameters of the subchondral bone plate and the subarticular spongiosa of defect region between three treatment groups.

Parameter	Unit	Adjacent	Defect			Overall *P*	Specific *P*		
			Debridement	Microfracture	Enhanced microfracture		*	#	§
Subchondral bone plate									
BMD	mg/cm^3^	631.74 ± 217.62	630.79 ± 85.34	410.01 ± 222.01^†^	460.05 ± 196.34^†^	0.066	0.07	0.148	0.885
BV/TV	%	78.32 ± 7.09	1.55 ± 0.26^†^	1.25 ± 1.23^†^	1.19 ± 0.95^†^	0.378	0.470	0.421	0.996
BS/BV	mm^−1^	50.83 ± 8.56	120.74 ± 12.55^†^	172.86 ± 76.16^†^	162.76 ± 21.37^†^	0.152	0.159	0.287	0.925
BS/TV	mm^−1^	39.48 ± 5.42	2.54 ± 1.81^†^	1.65 ± 1.44^†^	1.83 ± 1.33^†^	0.583	0.586	0.713	0.976
Ct.Th	mm	69.77 ± 16.92	0.10 ± 0.02	0.07 ± 0.02	0.06 ± 0.01	**0.006**	0.067	**0.005**	0.357
Subarticular spongiosa									
BMD	mg/cm^3^	696.45 ± 236.77	851.65 ± 32.64	572.59 ± 244.75	735.30 ± 244.95	0.129	0.112	0.385	0.682
BV/TV	%	43.03 ± 7.42	40.08 ± 2.13^†^	36.91 ± 3.90	46.07 ± 6.38	**0.010**	0.460	0.085	**0.008**
BS/BV	mm^−1^	29.79 ± 4.58	29.65 ± 2.59	28.25 ± 3.00	26.49 ± 3.47^†^	0.228	0.708	0.202	0.586
BS/TV	mm^−1^	12.58 ± 1.59	11.88 ± 1.16	10.44 ± 1.65^†^	12.08 ± 1.24	0.109	0.193	0.967	0.128
Tb.Th	mm	0.39 ± 0.82	0.12 ± 0.01^†^	0.12 ± 0.02	0.13 ± 0.02	0.380	0.765	0.447	0.853
Tb.Sp	mm	0.26 ± 0.36	0.18 ± 0.02	0.23 ± 0.05^†^	0.17 ± 0.02	**0.015**	0.093	0.622	**0.015**
Tb.Pf	mm^−1^	−2.02 ± 4.28	−0.01 ± 1.67	−2.17 ± 5.16	−5.40 ± 5.26^†^	0.229	0.891	0.223	0.426
Tb.N	mm^−1^	3.57 ± 0.99	3.46 ± 0.38	3.01 ± 0.50^†^	3.61 ± 0.53	0.108	0.266	0.847	0.108
SMI	−/−	0.66 ± 0.55	0.95 ± 0.32^†^	0.90 ± 0.65	0.49 ± 0.78^†^	0.378	0.988	0.410	0.491
DA	−/−	1.87 ± 0.16	0.42 ± 0.03	0.44 ± 0.03^†^	0.39 ± 0.03^†^	0.055	0.645	0.384	0.088
FD	−/−	2.40 ± 0.08	2.46 ± 0.07	2.43 ± 0.04	2.48 ± 0.02^†^	0.282	0.984	0.947	0.880

Values are expressed as mean ± SD. ^†^Significant difference between defect VOI and adjacent normal control structure detected with Mann-Whitney U test. For overall *P* values, one-way ANOVA test was performed. Specific *P* values were reported as follows: ^*^P < 0.05 for debridement *versus* microfracture; ^#^*P* < 0.05 for debridement *versus* enhanced microfracture; ^§^*P* < 0.05 for microfracture *versus* enhanced microfracture. Bold values indicate a significant difference between groups (*P* < 0.05). BMD, bone mineral density; BV/TV, bone volume fraction; BS/BV, specific bone surface; BS/TV, bone surface density; Ct.Th, cortical thickness; Tb.Th, trabecular thickness; Tb.Sp, trabecular separation; Tb.Pf, trabecular pattern factor; Tb.N, trabecular number; SMI, structure model index; DA, degree of anisotropy; FD, fractal dimension.
