# Supplementary material for: Early loss of subchondral bone following microfracture is counteracted by bone marrow aspirate in a translational model of osteochondral repair
Source: Sci Rep. 2017 Mar 27;7:45189. doi: 10.1038/srep45189 (PMC5366926; doi:10.1038/srep45189)
Supplement: Supplementary Materials [file srep45189-s1.pdf]

Supplementary Materials

Early loss of subchondral bone following microfracture is counteracted by bone marrow aspirate in a translational model of osteochondral repair

Liang Gao[1], Patrick Orth[1,2], Kathrin Müller-Brandt[1], Lars K. H. Goebel[1,2], Magali Cucchiarini[1], Henning Madry[1,2,*]

[1]Center of Experimental Orthopaedics, Saarland University, Homburg, Germany.

[2]Department of Orthopaedic Surgery, Saarland University Medical Center, Homburg, Germany

[*]Corresponding author

E-mail: henning.madry@uks.eu

List of Supplementary Materials

Supplementary Table S1. Macroscopic evaluation of the articular cartilage repair tissue.

Supplementary Table S2. Histological evaluation of the articular cartilage repair tissue.

Supplementary Table S3. Evaluation of immunoreactivity to type-II collagen in the articular cartilage repair tissue.

Supplementary Table S4. Comparison of micro-CT parameters of VOIs between the defect area and the adjacent control.

Supplementary Table S5. Change of bone volume fraction (BV/TV) of the subchondral bone plate under defects treated by the three treatment strategies at 4 weeks postoperatively.

Supplementary Table S6. Change of bone volume fraction (BV/TV) of the subarticular spongiosa under defects treated by the three treatment strategies at 4 weeks postoperatively.

Table S1. Macroscopic evaluation of the articular cartilage repair tissue.

Parameter	Debridement	Microfracture	Enhanced microfracture	Overall *P*	Specific *P*		
					*	#	§
Color of the repair tissue	2.60 ± 1.22	2.83 ± 0.75	2.33 ± 1.21	0.785	0.931	0.662	0.485
Blood vessels in the repair tissue	1.80 ± 2.05	3.00 ± 0.63	3.00 ± 1.10	0.271	0.537	0.329	1.000
Surface of the repair tissue	2.20 ± 1.79	2.00 ± 1.26	2.00 ± 1.26	0.966	0.792	0.931	0.937
Filling of the defect	2.00 ± 1.87	2.33 ± 0.82	1.50 ± 1.05	0.539	0.931	0.662	0.240
Degeneration of the adjacent cartilage	0.00 ± 0.00	0.50 ± 0.84	0.67 ± 0.82	0.300	0.429	0.177	0.699
Total	8.60 ± 6.31	10.67 ± 1.86	9.50 ± 4.46	0.745	1.000	1.000	0.394

Values are expressed as mean ± SD. Graded on a scale of 0 to 20 (20 = worst repair; 0 = complete regeneration) according to Goebel *et al.*[20]. No statistic significant difference exists for all parameters and the average total score among the three treatment groups. Overall *P* values were for comparisons among the three treatment groups. Specific *P* values were for comparisons of two of the three treatment groups as follows: $^{*}P < 0.05$ for debridement *versus* microfracture; $^{\#}P < 0.05$ for debridement *versus* enhanced microfracture; $^{\S}P < 0.05$ for microfracture *versus* enhanced microfracture group.

Table S2. Histological evaluation of the articular cartilage repair tissue.

Parameter	Debridement	Microfracture	Enhanced microfracture	Overall *P*	Specific *P*		
					*	#	§
Filling of defect	1.48 ± 1.22	1.90 ± 1.29	1.06 ± 0.73	0.415	1.000	0.662	0.093
Integration	1.25 ± 0.44	1.40 ± 0.61	1.50 ± 0.51	0.446	0.537	0.247	0.699
Matrix staining	2.95 ± 1.11	3.54 ± 0.50	3.44 ± 0.65	0.344	0.662	0.662	0.699
Cellular morphology	4.35 ± 1.00	4.54 ± 0.87	4.02 ± 1.08	0.595	0.429	0.792	0.310
Architecture (defect)	2.20 ± 0.99	2.35 ± 0.67	2.25 ± 0.60	0.896	0.537	0.662	0.699
Architecture (surface)	0.90 ± 0.90	0.54 ± 0.74	1.21 ± 1.17	0.261	0.662	0.537	0.065
Subchondral bone	1.28 ± 0.99	2.58 ± 1.01	1.69 ± 1.11	0.075	**0.050**	0.714	0.205
Tidemark	4.00 ± 0.00	2.98 ± 0.14	3.98 ± 0.14	0.670	0.662	0.662	1.000
Total	18.40 ± 3.51	20.83 ± 3.27	19.15 ± 1.52	0.314	0.247	0.662	0.093

Values are expressed as mean ± SD. Bold values indicate a significant difference between groups ($P < 0.05$). Graded on a scale of 0 to 31 (31 = no repair; 0 = complete regeneration) according to Sellers *et al.*[21]. No difference of the average total score and each parameter existed in the histological scoring of the repair tissue between the three treatment groups. Overall *P* values were for comparisons among the three treatment groups. Specific *P* values were for comparisons of two of the three treatment groups as follows: $^{*}P < 0.05$ for debridement *versus* microfracture; $^{\#}P < 0.05$ for debridement *versus* enhanced microfracture; $^{\S}P < 0.05$ for microfracture *versus* enhanced microfracture.

Table S3. Evaluation of immunoreactivity to type-II collagen in the articular cartilage repair tissue.

	Debridement	Microfracture	Enhanced microfracture	Overall *P*	Specific *P*		
					*	#	§
Point value	1.80 ± 1.48	0.70 ± 0.52	0.50 ± 0.55	0.072	0.177	0.126	0.699

Values are expressed as mean ± SD. Immunoreactivity of type-II collagen was compared with the adjacent hyaline cartilage and graded on a scale from 0 to 4 (0 = no immunoreactivity; 4 = stronger immunoreactivity than adjacent cartilage)[33]. No statistically significant differences existed between the three treatment groups. "Overall *P* values" were for comparisons among the three treatment groups, while "specific *P* values" referred to comparisons between two treatment groups as follows: $^{*}P < 0.05$ for debridement *versus* microfracture; $^{\#}P < 0.05$ for debridement *versus* enhanced microfracture; $^{\S}P < 0.05$ for microfracture *versus* enhanced microfracture.

Table S4. Comparison of micro-CT parameters of VOIs between the defect area and the adjacent control.

Parameter	Unit	Debridement		P_1	Microfracture		P_2	Enhanced microfracture		P_3
		Adjacent	Defect		Adjacent	Defect		Adjacent	Defect	
Subchondral bone plate										
BMD	mg/cm^3	772.33 ± 70.32	630.79 ± 85.34	0.225	546.46 ± 251.06	410.01 ± 222.01	**0.028**	599.87 ± 234.19	460.05 ± 196.34	**0.028**
BV/TV	%	78.71 ± 4.02	2.03 ± 1.19	**0.028**	80.35 ± 7.44	1.25 ± 1.23	**0.028**	75.97 ± 8.98	1.19 ± 0.95	**0.028**
BS/BV	mm^{-1}	45.89 ± 9.11	120.74 ± 12.55	**0.028**	50.39 ± 3.01	172.86 ± 76.16	**0.028**	55.39 ± 10.45	162.76 ± 21.37	**0.028**
BS/TV	mm^{-1}	35.88 ± 5.65	2.54 ± 1.81	**0.028**	40.43 ± 3.81	1.65 ± 1.44	**0.028**	41.53 ± 5.90	1.83 ± 1.33	**0.028**
Ct.Th	Mm	0.08 ± 0.03	0.10 ± 0.02	0.225	0.06 ± 0.01	0.07 ± 0.02	0.600	0.07 ± 0.01	0.06 ± 0.01	0.249
Subarticular spongiosa										
BMD	mg/cm^3	863.89 ± 63.23	851.65 ± 32.64	0.345	585.94 ± 268.01	572.59 ± 244.75	0.600	667.43 ± 246.31	735.30 ± 244.95	0.917
BV/TV	%	46.15 ± 6.27	40.08 ± 2.13	**0.046**	41.80 ± 5.65	36.91 ± 3.90	0.116	41.66 ± 9.96	46.07 ± 6.38	0.173
BS/BV	mm^{-1}	28.03 ± 3.77	29.65 ± 2.59	0.075	30.55 ± 12.62	28.25 ± 3.00	0.116	30.49 ± 5.46	26.49 ± 3.47	**0.046**
BS/TV	mm^{-1}	12.81 ± 1.44	11.88 ± 1.16	0.173	12.62 ± 1.61	10.44 ± 1.65	**0.028**	12.35 ± 1.94	12.08 ± 1.24	0.463
Tb.Th	Mm	0.74 ± 1.38	0.12 ± 0.01	**0.043**	0.11 ± 0.02	0.12 ± 0.02	0.075	0.37 ± 0.64	0.13 ± 0.02	0.463
Tb.Sp	Mm	0.46 ± 0.67	0.18 ± 0.02	0.463	0.17 ± 0.02	0.23 ± 0.05	**0.046**	0.17 ± 0.03	0.17 ± 0.02	0.345
Tb.Pf	mm^{-1}	-2.19 ± 2.16	-0.01 ± 1.67	0.917	-2.10 ± 4.10	-2.17 ± 5.16	0.917	-1.80 ± 6.16	-5.40 ± 5.26	**0.046**
Tb.N	mm^{-1}	3.19 ± 1.68	3.46 ± 0.38	0.345	3.81 ± 0.59	3.01 ± 0.50	**0.028**	3.64 ± 0.57	3.61 ± 0.53	0.917
SMI	-/-	0.47 ± 0.31	0.95 ± 0.32	**0.046**	0.69 ± 0.58	0.90 ± 0.65	0.345	0.80 ± 0.70	0.49 ± 0.78	**0.043**
DA	-/-	1.84 ± 0.18	0.42 ± 0.03	0.093	1.90 ± 0.14	0.44 ± 0.03	**0.028**	1.86 ± 0.17	0.39 ± 0.03	**0.028**
FD	-/-	2.42 ± 0.06	2.41 ± 0.07	0.249	2.41 ± 0.09	2.43 ± 0.04	0.600	2.38 ± 0.08	2.48 ± 0.02	**0.046**

Values are expressed as mean ± SD. Mann-Whitney U test was applied for comparison of parameters of subchondral bone between adjacent and defect regions. Bold values indicate a significant difference between groups (P < 0.05). BMD, bone mineral density; BV/TV, bone volume fraction; BS/BV, specific bone surface; BS/TV, bone surface density; Ct.Th, cortical thickness; Tb.Th, trabecular thickness; Tb.Sp, trabecular

separation; Tb.Pf, trabecular pattern factor; Tb.N, trabecular number; SMI, structure model index; DA, degree of anisotropy; FD, fractal dimension.

Table S5. Change of bone volume fraction (BV/TV) of the subchondral bone plate under defects treated by the three treatment strategies at 4 weeks postoperatively.

Treatment	BV/TV (%)			Preservation rate (%)	Overall *P*	Specific *P*		
	SBP-adjacent	SBP-defect$_{(calculated)}$	SBP-defect$_{(measured)}$			*	#	§
Debridement	78.71 ± 4.02	78.71 ± 4.02	1.55 ± 0.26	1.97 ± 0.33				
Microfracture	80.35 ± 7.44	58.66 ± 5.43	1.25 ± 1.23	2.12 ± 2.51	0.966	0.429	1.000	0.818
Enhanced microfracture	75.97 ± 8.98	55.46 ± 6.56	1.19 ± 0.95	2.14 ± 1.59				

Bone volume fraction (BV/TV) of SBP-adjacent and SBP-defect [SBP-defect$_{(measured)}$] were directly calculated with CTAnalyzer software, and expected BV/TV of SBP-defect [SBP-defect$_{(calculated)}$] was calculated according to Formulas (1) and (3). Preservation rate was computed as the ratio of BV/TV of SBP-defect$_{(measured)}$ to BV/TV of SBP-defect$_{(calculated)}$. Overall *P* values were calculated with one way ANOVA for comparison of the preservation rate of the calculated BV/TV among the three treatment groups, while specific *P* values were analyzed with Mann Whitney U test and reported as follows: $^{*}P < 0.05$ for preservation rate of BV/TV of SBP-defect$_{(calculated)}$ between debridement and microfracture group; $^{\#}P < 0.05$ for preservation rate of BV/TV of SBP-defect$_{(calculated)}$ between debridement and enhanced microfracture group; $^{\S}P < 0.05$ for preservation rate of BV/TV of SBP-defect$_{(calculated)}$ between microfracture and enhanced microfracture group.

Table S6. Change of bone volume fraction (BV/TV) of the subarticular spongiosa under defects treated by the three treatment strategies at 4 weeks postoperatively.

Treatment	Bone volume fraction (BV/TV) (%)			Preservation rate (%)	Overall *P*	Specific *P*		
	SAS-adjacent	SAS-defect $_{(calculated)}$	SAS-defect $_{(measured)}$			*	#	§
Debridement	46.15 ± 6.27	46.15 ± 6.27	40.08 ± 2.13	86.85 ± 12.78				
Microfracture	41.80 ± 5.65	30.51 ± 4.12	36.91 ± 3.90	122.54 ± 18.98	0.0002	0.009	0.004	0.015
Enhanced microfracture	41.66 ± 9.96	30.41 ± 7.27	46.07 ± 6.38	155.72 ± 24.51				

Bone volume fraction (BV/TV) of SAS-adjacent and SAS-defect [SAS-defect$_{(measured)}$] were directly calculated with CTAnalyzer software, and expected BVTV of SAS-defect [SAS-defect$_{(calculated)}$] was calculated according to Formulas (2) and (4). Preservation rate was computed as the ratio of BV/TV of SAS-defect $_{(measured)}$ to BV/TV of SAS-defect$_{(calculated)}$. Overall *P* values were calculated with one way ANOVA for comparison of the preservation rate of SAS-defect$_{(calculated)}$ among the three treatment groups, while specific *P* values were analyzed with Mann-Whitney U test and reported as follows: $^{*}P < 0.05$ for preservation rate of BV/TV of SAS-defect(calculated) of defects from debridement versus microfracture group; $^{\#}P < 0.05$ for preservation rate of BV/TV of SAS-defect(calculated) of defects from debridement *versus* enhanced microfracture group; $^{\S}P < 0.05$ for preservation rate of BV/TV of SAS-defect(calculated) of defects from microfracture *versus* enhanced microfracture group.
